# The synergistic effect of electroacupuncture and bone mesenchymal stem cell transplantation on repairing thin endometrial injury in rats

**DOI:** 10.1186/s13287-019-1326-6

**Published:** 2019-08-07

**Authors:** Liangjun Xia, Qingyu Meng, Jin Xi, Qin Han, Jie Cheng, Jie Shen, Youbing Xia, Liyun Shi

**Affiliations:** 1 School of Medicine and Life Science, Nanjing University of Chinese Medicine, Nanjing, 210046 China; 2 Xuzhou Medical University, Xuzhou, 221004 China; 3 The Second Clinical College, Nanjing University of Chinese Medicine, Nanjing, 210046 China

**Keywords:** Bone marrow mesenchymal stem cells, Thin endometrium, Electroacupuncture treatment, Stem cell migration

## Abstract

**Background:**

Tissue regeneration disorder after endometrial injury is an important cause of intrauterine adhesions, amenorrhea, and infertility in women. Both bone marrow mesenchymal stem cell (BMSC) transplantation and electroacupuncture (EA) are promising therapeutic applications for endometrial injury. This study examined their combined effects on thin endometrium in rats and the possible mechanisms underlying these effects.

**Methods:**

A thin endometrial model was established in Sprague-Dawley (SD) rats by perfusing 95% ethanol into the right side of the uterus. The wounds were randomly treated with PBS (model group), BMSCs only (BMSC group), EA only (EA group), and BMSCs combined with EA (BMSC + EA group). Endometrial morphological alterations were observed by hematoxylin and eosin (H&E) staining. Changes in markers of epithelial and stromal endometrium cells, endometrial receptivity-related chemokines, and paracrine factors were detected using immunohistochemistry, western blotting, and quantitative reverse-transcription polymerase chain reaction (qRT-PCR). Finally, the functional recovery of the uterus was evaluated by determining the rate of embryo implantation.

**Results:**

As shown by endometrial morphology, the damaged uteri in all the treatment groups recovered to some extent, with the best effects observed in the BMSC + EA group. Further studies showed that EA promoted the migration of transplanted BMSCs to damaged uteri by activating the stromal cell-derived factor-1/C-X-C chemokine receptor type 4 (SDF-1/CXCR4) axis. As compared with the other groups, upregulated expression of endometrial cytokeratin and vimentin, increased secretion of vascular endothelial growth factor (VEGF) and basic fibroblast growth factor (bFGF) in endometrial lesions, and improved embryo implantation rates on the 8th day of pregnancy were found in the BMSC + EA group.

**Conclusions:**

EA plays an important role in supporting BMSCs in the repair of thin endometrium, most likely by promoting the migration of BMSCs and enhancing the paracrine effect of BMSCs.

**Electronic supplementary material:**

The online version of this article (10.1186/s13287-019-1326-6) contains supplementary material, which is available to authorized users.

## Background

A thin endometrium refers to an intimal thickness of < 7 mm before ovulation or on the day of human chorionic gonadotropin administration [1]. Excessive uterine curettage, endocrine disorders, endometrial tuberculosis, and other factors can lead to endometrial thinning and growth restriction [2, 3]. A thin endometrium may also result from endometrial basal vascular dysplasia [4]. A thin endometrium has implications for a woman’s fertility, as it is related to lower implantation and pregnancy rates. In terms of prevalence, a thin endometrium affects about one in three to two in three women undergoing in-vitro fertilization and embryo transfer (IVF-ET) [5].

A number of treatments have been tried to increase endometrial development. These include intra-uterine granulocyte colony-stimulating factor, extended estrogen support, human chorionic gonadotropin priming in the follicular phase, and drugs that increase endometrial blood flow (e.g., pentoxyfilline, tocopherol, sildenafil, and l-arginine) [6]. To date, none of these treatments have been validated. Thus, finding a successful treatment for a thin endometrium remains a challenge.

Mesenchymal stem cells (MSCs) have various qualities that make them the ideal choice for cell/gene therapy and regenerative medicine, including therapies targeting the uterus [7, 8]. These qualities include ease of separation from various tissues; plentiful proliferation capacity in vitro, without any change in their biological features; tropism to damaged tissue; weakly immunogenicity; and secretion of anti-inflammatory molecules. Furthermore, they pose no risk to normal cells/tissues. Following an injection of bone mesenchymal stem cells (BMSCs) into the body, these cells secrete various growth factors in the endometrium, thereby effectively promoting the proliferation, migration, and differentiation of microvascular endothelial cells [9, 10]. Previous research has reported the upregulation of endometrial receptivity markers in a murine thin endometrium model, in which mice treated with BMSCs showed moderate improvements in fertility [11]. Clinical studies have confirmed that BMSCs isolated from autologous peripheral blood aided endometrial reconstruction and restored normal menstruation in patients with endometrial injury and that some patients became pregnant without any medical intervention after treatment [12]. Although stem cells can repair endometrial damage to a certain extent, due to the influence of the intrauterine environment, there are individual differences in the role of stem cells in the uterine cavity. Thus, their efficacy is unstable.

In recent years, a number of studies have demonstrated the effectiveness of electroacupuncture (EA) in the field of assisted reproduction [13–15]. Some studies have reported that EA regulated the uterine microcirculation [16], upregulated the expression of the estrogen receptor (ER) and progesterone receptor (PR) in the endometrial surface, increased serum estrogen levels, promoted endometrial regeneration [17], improved clinical pregnancy and/or live birth rates [18], and relieved pain and anxiety during embryo transfer [19]. Other studies have shown that EA stimulated the proliferation and activation of stem cells and further promoted the stem cell migration to injured areas following tissue damage [20, 21]. Based on previous studies on the effects of EA on endometrial and stem cells, we aimed to explore the potential beneficial role of EA on BMSCs in the uterus. With this aim in mind, we investigated the combined effects of BMSC transplantation and EA on thin endometrium of rats and the possible mechanisms underlying these effects.

## Materials and methods

### Animals

Mature male and female Sprague-Dawley (SD) rats aged 8–12 weeks (weight 200–240 g) and male SD rats aged 3 weeks (weight 50 g) were obtained from a specific pathogen-free (SPF)-level facility (Beijing Vital River Experimental Animal Technology, China). They were kept under a controlled 12:12-h light–dark cycle, with a standard diet and free access to water in the Animal Experimental Center of Nanjing University of Traditional Chinese Medicine. The study protocol was approved by the Animal Care and Use Committee of Nanjing University of Chinese Medicine on 27 April 2018 (reference number: 201804A021).

### Groups and treatments

The study comprised of 100 rats, which were randomly assigned to the following treatment groups (*n* = 20 for each group): control, phosphate-buffered saline (PBS; model group), BMSCs only (BMSC group), EA only (EA group), and BMSCs combined with EA (BMSC + EA group). In accordance with Gao et al.’s protocol, the right uterus in the model, BMSC, EA, or BMSC + EA group was injected with 95% ethanol to establish a thin endometrial model (left uterus maintained as a control) [22]. On the 1st, 3rd, and 7th days of the study, the rats in the model group were injected with 1 mL PBS through the tail vein; the rats in the BMSC group were injected with 1 mL of 1 × 10^6^ cells/mL BMSC suspension through the tail vein; in the EA group, EA was administered for 15 min each day (beginning at 10:00 am) after the establishment of the thin endometrial model and continued for three estrous cycles; in the EA + BMSC group, on the 1st, 3rd, and 7th days of the study, BMSCs were injected through the tail vein before receiving the EA treatment. On the day of the third estrus, the rats in each group were sacrificed and the uteruses were harvested.

### EA stimulation

All the animals were anesthetized with ketamine. In the EA and BMSC + EA groups, EA stimulation was applied to the acupuncture points of unilateral Sanyinjiao (SP6), Guanyuan (CV4), and Zigong (EX-CA1) using an EA stimulator instrument (Model SDZ-II; Suzhou Medical Appliance Factory, Suzhou, China). In the control, model, and BMSC groups, the rats were not treated with EA. The depth and location of the EA, atlas of the skeleton, acupoints of the animals, and anatomical locations were as described in the Handbook of Practical Animal Acupuncture [23]. SP6 was situated about 10 mm above the top of the medial malleolus and on the posterior border of the tibia. CV4 was located at a point of 3/5 down the ventral midline, connecting the umbilicus to the pubic tubercle. EX-CA1 was located about 20 mm below the umbilical cord and 10 mm beside the midline of the abdomen. Two 0.3-mm stainless steel acupuncture needles (Wuxi Jiajian Medical Devices Co., Ltd., Wuxi, China) connected to an output terminal were inserted at a depth of 2 mm into the abovementioned acupuncture points. Stimulation by disperse-dense waves with 2/15 Hz frequencies was generated at an intensity of muscle twitch threshold and administered for 15 min per day continued three estrous cycles.

### BMSCs culture in vitro

BMSCs were isolated from the bone marrow of 3-week-old male SD rats. Briefly, femurs and tibiae were collected from immature rats sacrificed by cervical dislocation. Bone marrow cells were harvested by flushing the marrow cavity with low glucose Dulbecco’s modified Eagle’s medium (L-DMEM) (Gibco). The suspended cells were then collected by centrifugation at 1500 rpm for 5 min. The cells were resuspended and cultured in L-DMEM media with 10% fetal bovine serum (FBS) and 1% penicillin-gentamicin at 37 °C in a humidified incubator with 5% carbon dioxide. Nonadherent hematopoietic cells were removed after 24 h. The culture medium was changed every 2–3 days. Adherent BMSCs were harvested by 0.25% trypsin-ethylenediaminetetraacetic acid (trypsin/EDTA) (Gibco) when reaching 80–90% confluence. BMSCs cultured in the third passage were used for transplantation. The cells were harvested with trypsin/EDTA, washed twice with PBS, and resuspended at a concentration of 1 × 10^6^ cells/mL in 1 mL of PBS.

### Phenotypic identification of BMSCs

Before in vivo experiments, the mesenchymal phenotype of the cells was assessed by fluorescence-activated cell sorting (FACS). The cells in the third passage were harvested with 0.25% trypsin/EDTA, resuspended at a concentration of 1 × 10^6^ cells/mL in PBS, and separated in a microcentrifuge tube. According to the instructions of the manufacturer, corresponding doses of CD45, CD29, and CD90 monoclonal antibodies were added to each tube in turn. Meanwhile, the same type of negative control was established. Then, it was mixed well and incubated at 4 °C for 1 h and detected by flow cytometry.

### Cell labeling with CM-Dil

To track the transplanted cells in vivo, the BMSCs were labeled with CM-Dil (C7000, Invitrogen, USA). Briefly, BMSCs were made into a single cell suspension in L-DMEM medium containing 10% FBS. Then, 1 mL of CM-Dil reagent was added, mixed using a pipette, and incubated for 5 min at room temperature. Subsequently, 2 mL of FBS was added to stop the staining. After centrifugation, the supernatant was discarded and cells were resuspended in fresh medium. The above staining procedure was repeated three times. The cells were then resuspended in PBS and transplanted into the rat through the tail vein.

### Hematoxylin-eosin(H&E) staining

H&E staining was performed as previously described [24]. After fixation in 4% paraformaldehyde, the uterine horns were embedded in paraffin, and 4-μm serial sections were produced. The sections were mounted on slides and immersed in xylene (10 min, twice), and rehydrated in a decreasing ethanol series diluted in distilled water (100%, 100%, 95%, 90%, 80%, and 70%, 1 min each). The sections were then rinsed in deionized water, stained in hematoxylin for 80 s, rinsed in deionized water, and finally stained in eosin for 3 s. After the color reaction, the sections were dehydrated through an ethanol series in xylene and mounted using Permount TM Mounting Medium (Thermo Fisher Scientific). Sections were taken using an IX73 microscope (Olympus Corporation, Shinjuku, Tokyo, Japan). Image J (Image in Java, National Institute of Health, Bethesda, MD, USA) software was used to determine the thickness of the endometrium (the vertical distance from the endometrial junction to the myometrium to the uterine cavity) and calculate the number of blood vessels and glands in four different fields of view, with their averages recorded.

### Immunohistochemistry (IHC)

The sections were deparaffinized in xylene, rehydrated through a series of ethanol washes, and rinsed in PBS. Endogenous peroxidase activity was blocked by incubating the sections in 0.3% hydrogen peroxide (H_2_O_2_) in methanol for 10 min at room temperature. Sections with 1× sodium citrate antigen repair solution were boiled in a microwave at high heat for 1 min, medium heat for 2 min, and low heat for 7 min, and then allowed to cool at room temperature. They were then washed in PBS and punched with solution (0.5% (v/v) Tween-20 and 0.5% (v/v) Triton-100 in PBS) for 15 min at room temperature. Nonspecific binding sites were blocked with 1% bovine serum albumin in PBS for 1 h at room temperature and subsequently incubated with the rabbit polyclonal antibody against cytokeratin (1:200, sc-398871, Santa Cruz Biotechnology, USA) and mouse monoclonal antibody against vimentin (1:200, sc-73259, Santa Cruz Biotechnology, USA) overnight at 4 °C. After incubation with the primary antibody, the sections were washed with phosphate-buffered saline Tween-20 (PBST) and treated with anti-rabbit and anti-mouse immunoglobulin G (IgG) secondary antibodies. Protein expression was visualized with diaminobenzidine (Dako Cytomation, Carpinteria, CA, USA) staining. The reaction was stopped with distilled water, stained with hematoxylin, and dehydrated before mounting. The images were digitized by fluorescence microscopy on an IX73 microscope (Olympus Corporation). Finally, each tissue section was photographed under a high-power microscope, and the images were saved in TIFF format. Four fields of view were randomly selected from each uterine slice. The mean optical density (MOD) value of each slice was measured by image analysis software Image-Pro Plus 6.0 (Image Processing and Analysis in Java, National Institute of Health, Bethesda, MD, USA), and the expression intensity of the protein was semi-quantitatively compared using the average optical density method.

### Immunofluorescence staining

Uterine tissues were fixed in 4% paraformaldehyde for 24 h and then placed in a 15%, 30% sucrose solution to dehydrate at 4 °C. The dehydrated tissues were embedded in optimal cutting temperature (OCT) compound, rapidly frozen, and cut into 6-μm slices. The distribution of BMSCs stained with CM-Dil in rat uteri was observed by fluorescence microscopy on an IX73 microscope (Olympus Corporation). Four fields of view were randomly selected from each uterine section. The number of cells positive for fluorescence in each field was counted, and the average value was compared. The scale of the picture magnified × 200 represents 100 μm.

### Western blotting

The rat endometrium was scraped on ice, and the tissue was lysed in radio-immunoprecipitation assay (RIPA) buffer, supplemented with the protease inhibitors aprotinin (1 μg/mL) and phenylmethylsulphonyl fluoride (1 mM) and then homogenized using an ultrasonic crusher. After standing on ice for 1 h, the lysate was centrifuged at 4 °C for 15 min at 12,000*g*, and the supernatant was collected. The protein concentration was determined using a bicinchoninic acid (BCA). After boiling the uterus lysates for 10 min, 50 μg of protein from each sample was loaded onto a sodium dodecyl sulfate (SDS) polyacrylamide gel, and electrophoresis was performed at 90 V for 2 h. The separated proteins were then transferred onto polyvinylidene fluoride (PVDF) membranes (Millipore, Billerica, MA, USA) in transfer buffer (20% (*v*/*v*) methanol; 0.19 M glycine; 0.025 M Tris-Base, pH = 8.3) for an additional 80 min at 275 mA in ice water. Consequently, the blots were blocked with 5% bovine serum albumin in tris-buffered saline Tween-20 (TBST) for 1 h at room temperature. The following primary antibodies were used: rabbit polyclonal anti-C-X-C chemokine receptor type 4 (CXCR4) (YM3546; Immunoway, USA) diluted at 1:1000; rabbit polyclonal anti-stromal cell-derived factor-1 (SDF-1) (3530 s; CST, USA) diluted at 1:1000; rabbit polyclonal anti-basic fibroblast growth factor (anti-bFGF) (YT5549; Immunoway, USA) diluted at 1:1000; rabbit polyclonal anti-vascular endothelial growth factor (anti-VEGF) (ab11939; Abcam, UK) diluted at 1:1000; rabbit polyclonal anti-leukemia inhibitory factor (anti-LIF) (ab113262; Abcam, UK) diluted at 1:1000; and mouse monoclonal anti-homeobox A10 (anti-HoxA10) (sc-271428; Santa Cruz Biotechnology, USA) diluted at 1:1000. Mouse monoclonal anti-glyceraldehyde 3-phosphate dehydrogenase (anti-GAPDH) (51332S; CST, USA) was diluted at 1:3000 and incubated as the internal control. The membranes were finally rinsed with TBST and incubated with anti-rabbit and anti-mouse secondary antibodies (1:4000) for 1 h at room temperature, and the bands were visualized by using the Tanon-5200 gel imaging system (Shanghai, CHN). The expression levels of the protein bands were qualitatively determined using Image J software (National Institutes of Health, Bethesda, MD, USA), and the value was expressed as a ratio of the target protein to GAPDH.

### Quantitative reverse-transcription polymerase chain reaction (qRT-PCR)

Total RNA was extracted from the harvested uteri using the Trizol reagent (Gibco), and 1 μg of total RNA was subjected to reverse transcription of mRNA using oligo dT as a primer and a reverse transcription kit (Transgen Biotech, Beijing, China) to generate total cDNA. A quantitative PCR was then carried using the primers shown in Table 1 and FastStart Universal SYBR Green Master (Toyobo, Osaka, Japan) with a 7500 Real-Time PCR System (Applied Biosystems, Foster City, CA, USA). R-actin was used for normalization. The quantitative expression level was analyzed using the 2^−ΔΔCt^ method.

**Table 1:** Primers for real-time PCR

Gene name	Primer sequence
Rat-SDF-1-qPCR-F	GCATCAGTGACGGTAAGCCAG
Rat-SDF-1-qPCR-R	ATCTGAAGGGCACAGTTTGGAG
Rat-CXCR4-qPCR-F	AGCATTGCCATGGAAATATACACT
Rat-CXCR4-qPCR-R	AAATAGATGGTGGGCAGGAAGA
Rat-LIF- qPCR-F	CCTTCCCATCACCCCTGTAA
Rat-LIF- qPCR-R	CTGTGTAATAGGAAATAAAGAGGGC
Rat-HoxA-10- qPCR-F	TGGAAGCGTGGACATTCAGGT
Rat-HoxA-10- qPCR-R	GACCTTGGGCAAACGGGAA
Rat-VEGF- qPCR-F	ACAGTGAACGCTCCAGGATTTA
Rat-VEGF- qPCR-R	GCAGACCAAAGAAAGATAGAACAAAG
Rat-bFGF- qPCR-F	AGCGACCCACACGTCAAACTA
Rat-bFGF- qPCR-R	GGACTCCAGGCGTTCAAAGA
Rat-Actin- qPCR-F	GCGAGTACAACCTTCTTGCAGC
Rat-Actin- qPCR-R	ACCCATACCCACCATCACACC

### Fertility testing

Endometrial receptivity was assessed by testing the endometrial capacity to receive fertilized ova and retain embryos for pregnancy. The vaginal smear method was used to determine the estrous cycles of the rats. The female rats were mated at 1:1 ratio with sexually mature male rats on the day of estrus, and the presence or absence of vaginal plugs on the following morning was observed. Eight days after the appearance of vaginal plugs, the numbers and locations of embryos in each group were recorded.

### Statistical analysis

The SPSS statistical program (version 22) was used to analyze the data. Data were presented as the mean ± standard error (SEM). A one-way analysis of variance test was used to compare the means of normally distributed parameters (Levene’s test with 0.1 as the standard test). When equal variances were assumed, the least significant difference test was selected. Otherwise, Dunnett’s T3 test was carried out. A value of *P* < 0.05 was considered significant.

## Results

### In vitro culture and phenotypic identification of BMSCs

**Fig. 1:**
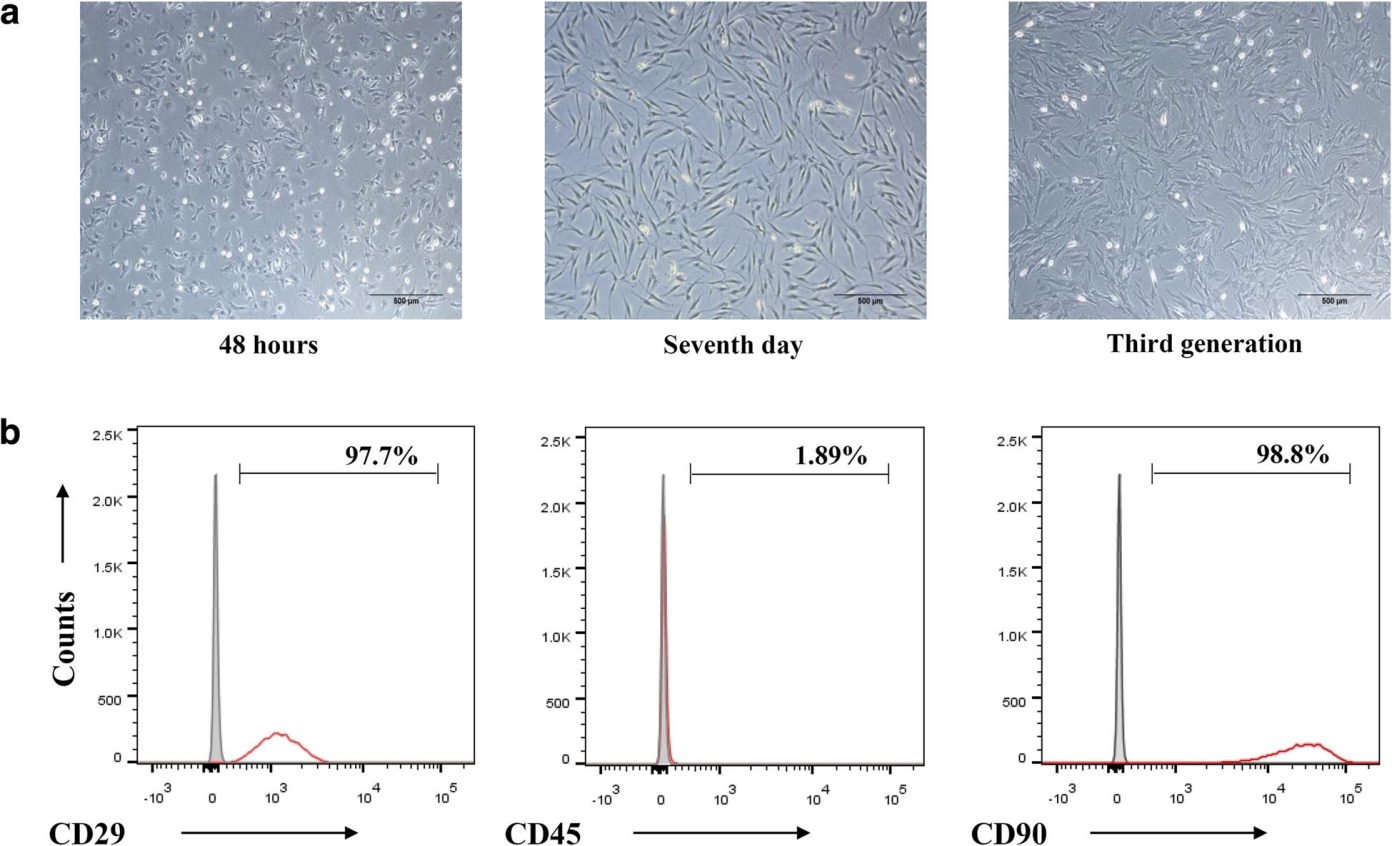
In vitro culture of SD rat BMSCs. **a** Morphological changes in BMSCs of rats at different culture times. **b** Staining of CD29, CD45, and CD90 detected by flow cytometry

The BMSCs obtained from rat bone marrow aspirates of 3-week-old rats were grown in culture as previously reported [25]. The cells showed small protrusions 48 h later. After 7 days, the cells became longer, closely attached to each other, and spread around. When the cell grew to the third generation, the growth rate increased rapidly, with most of the cells having a long spindle shape and uniform morphology, forming a cluster of spiral colony cells (Fig. 1a). As shown by the FACS analysis, the expression of surface markers CD29 and CD90 of BMSCs was 97.7% and 98.8%, respectively, whereas the expression of the hematopoietic stem cell marker CD45 was 1.89% (Fig. 1b).

### Morphological changes in the endometrium

**Fig. 2:**
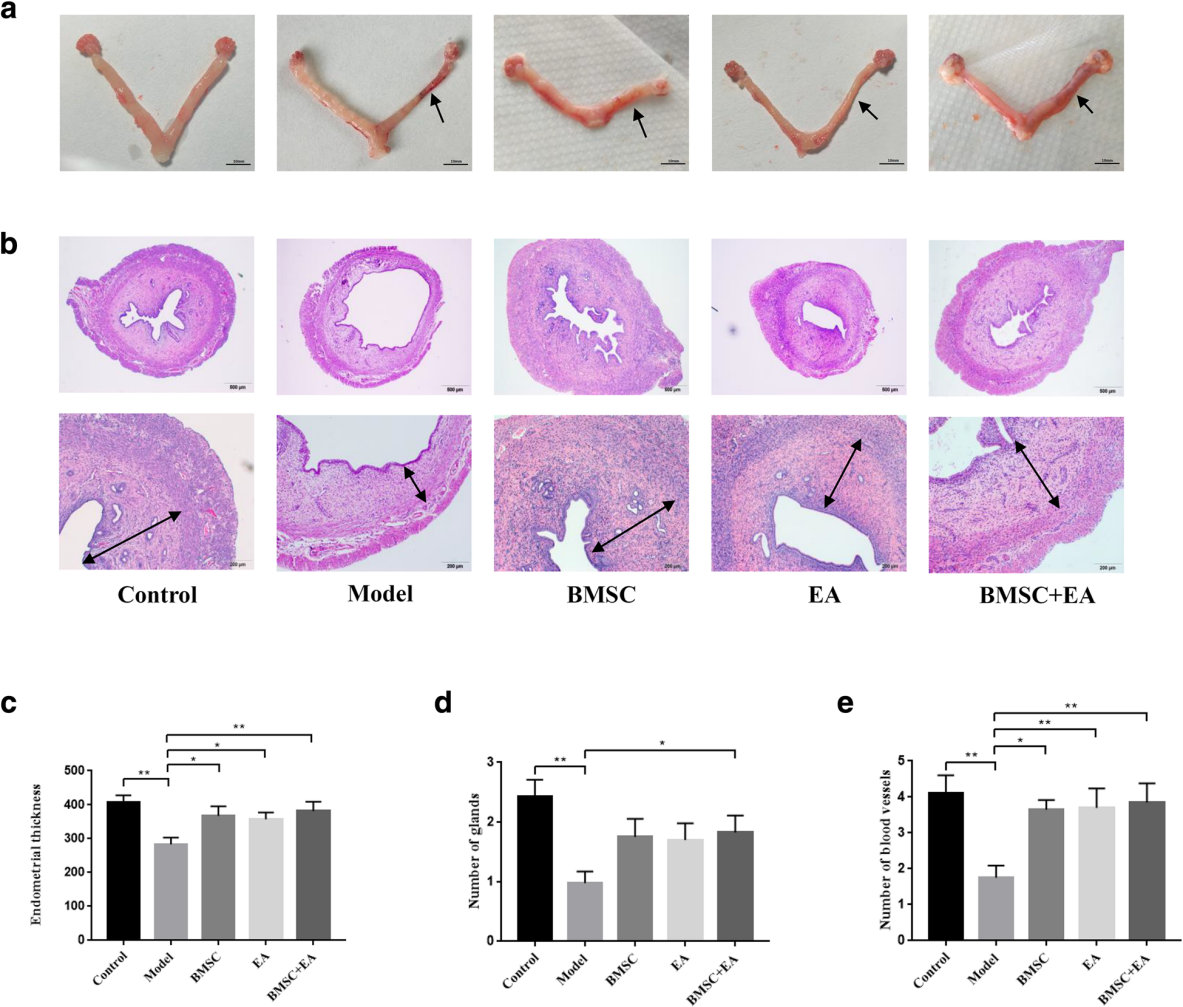
Uterine morphological features and changes. **a** The uterus specimen, with the arrow denoting the uterus after endometrial injury. **b** H&E staining of rat uterine tissue (× 40, × 100). **c** Endometrial thicknesses in each group. **d** The numbers of glands in each group. **e** The numbers of blood vessels in each group. Bars represent the mean ± standard error (SEM); *n* = 10 per group. *P < 0.05, **P < 0.01

As shown in Fig. 2a, after modeling, blood stasis appeared in the uterus of the model group, the lumen became thinner, and the original intact structure was lost. In the other groups (BMSC, EA, and BMSC + EA), the symptoms of blood stasis in the uterus were alleviated. The lumens were also thickened in these groups. In addition, the endometrial structure in the BMSC + EA group was most similar to that in the control group. From the H&E stained images, the endometrium structure in the control group was intact, the epithelial cells were closely arranged, and the blood vessels and glands were clearly visible. In the model group, the endometrium was severely damaged, glands and blood vessels were scarce, and severe endometrium thinning was apparent. As compared with the model group, the endometrial thicknesses in the BMSC, EA, and BMSC + EA groups (*P* < 0.05, *P* < 0.05, *P* < 0.01) increased significantly. The number of endometrial glands and blood vessels in the three intervention groups also increased. Notably, the endometrial layer of the BMSC + EA group was relatively intact. As compared with the other groups, the BMSC + EA group also contained more endometrial glands and capillaries and thicker endometrium (Fig. 2b–e; Additional file 1: Table S1).

### EA promotes migration of BMSCs to injured endometrial sites

**Fig. 3:**
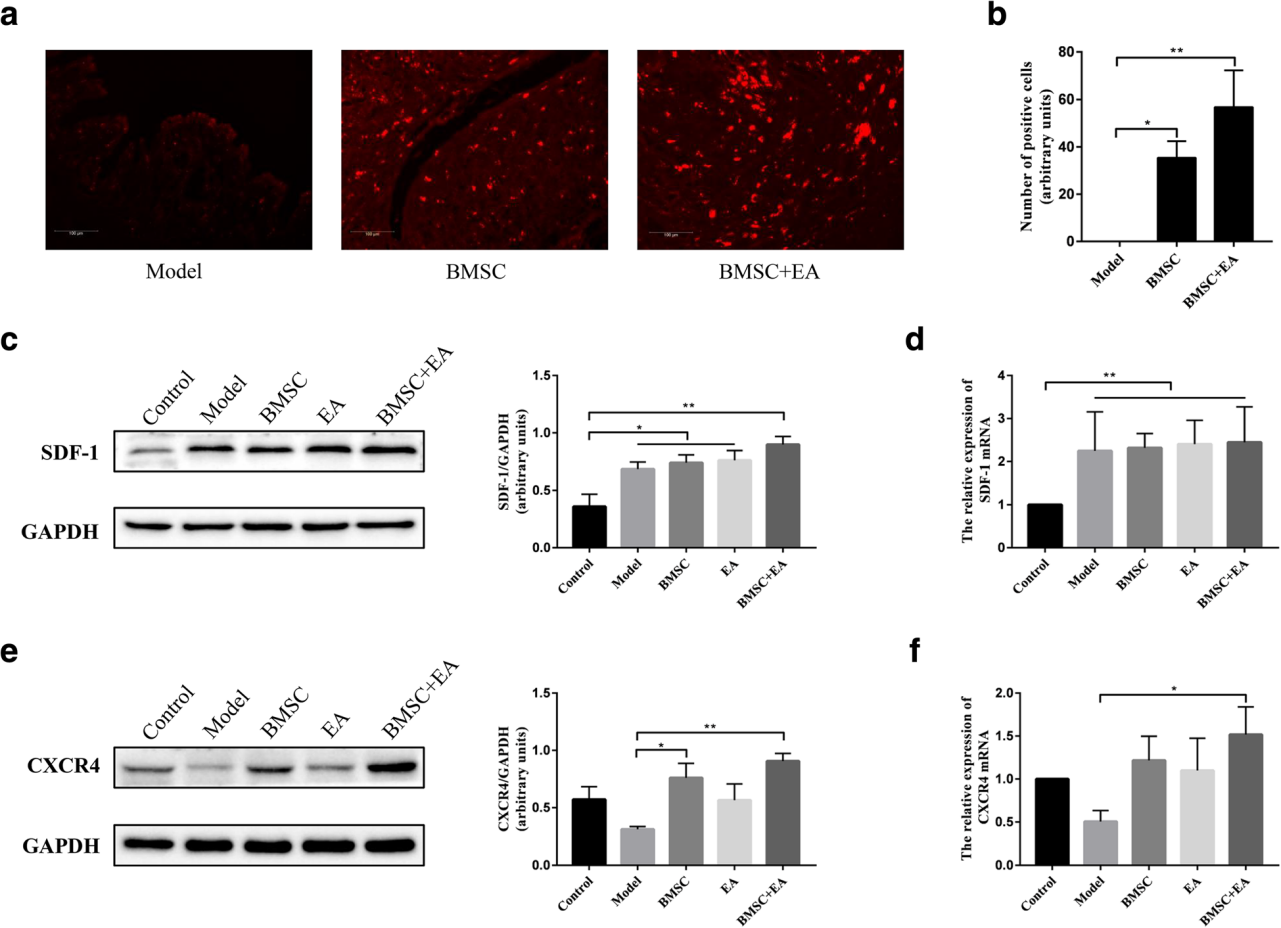
Effects of BMSC transplantation and EA on the release of endometrial surface chemokines. **a** Fluorescence microscopy was used to detect CM-Dil-positive BMSCs in the uterus of a thin endometrial rat model (× 200), *n* = 5 per group. **b** The histogram shows the number of positive cells per unit area of the endometrium. **c** The expression of stromal cell-derived factor-1 (SDF-1) protein in each group was detected by western blotting. The histogram shows the relative ratio of the expression level of the target protein to that of an internal reference. **d** SDF-1 mRNA expression in each group was detected by qRT-PCR. R-actin served as an internal reference for the qRT-PCR. **e** The expression of C-X-C chemokine receptor type 4 (CXCR4) protein in each group was detected by western blotting. The histogram shows the relative ratio of the expression level of the target protein to that of an internal reference. **f** CXCR4 mRNA expression in each group was detected by qRT-PCR. R-actin served as an internal reference for the qRT-PCR. Bars represent the mean ± standard error (SEM); *n* = 5 per group. *P < 0.05, **P < 0.01

To detect whether the transplanted BMSCs migrate to damaged endometrial tissue to aid tissue regeneration, the BMSCs were labeled with CM-Dil fluorescent dye before transplantation, and fluorescent cells were then tracked in vivo. After three estrus cycles, the CM-Dil-labeled cells were detected in the endometrium of the BMSC group and BMSC + EA group. The average number of fluorescent cells per unit area in the BMSC group and BMSC + EA group was 35.40 ± 7.03 and 56.80 ± 15.44, respectively. After stimulation with EA, more BMSCs accumulated in the damaged endometrium (Fig. 3a, b).

Chemokines are cytokines that have a low molecular weight and promote the migration of different cell types, including stem cells, through chemotactic mechanisms [26, 27]. Stem cells generally express the chemokine CXCR4 on their surface, which interacts with SDF-1 [28–30]. To investigate the role of EA in promoting the accumulation of BMSCs in the uterus, we examined the expression of SDF-1/CXCR4 on the endometrial surface. The expression of SDF-1 was upregulated after endometrial injury, with significantly strengthened expression in the BMSC + EA group (Fig. 3c, d). These findings indicate that the effect of EA on BMSC migration to the site of injury may be related to elevated expression of SDF-1 in damaged endometrial tissues. Then, we examined the expression of CXCR4 in each group. As shown in Fig. 3e and f, the expression of CXCR4 in the BMSC group or BMSC + EA group was upregulated compared with the model group, and upregulation in the combined group was significantly enhanced. Based on these results, we suggest that the presence of increased numbers of BMSCs in endometrial tissue may be associated with enhanced chemotaxis of BMSCs following EA stimulation.

### BMSCs combined with EA promotes endometrial cell regeneration and growth factor secretion

**Fig. 4:**
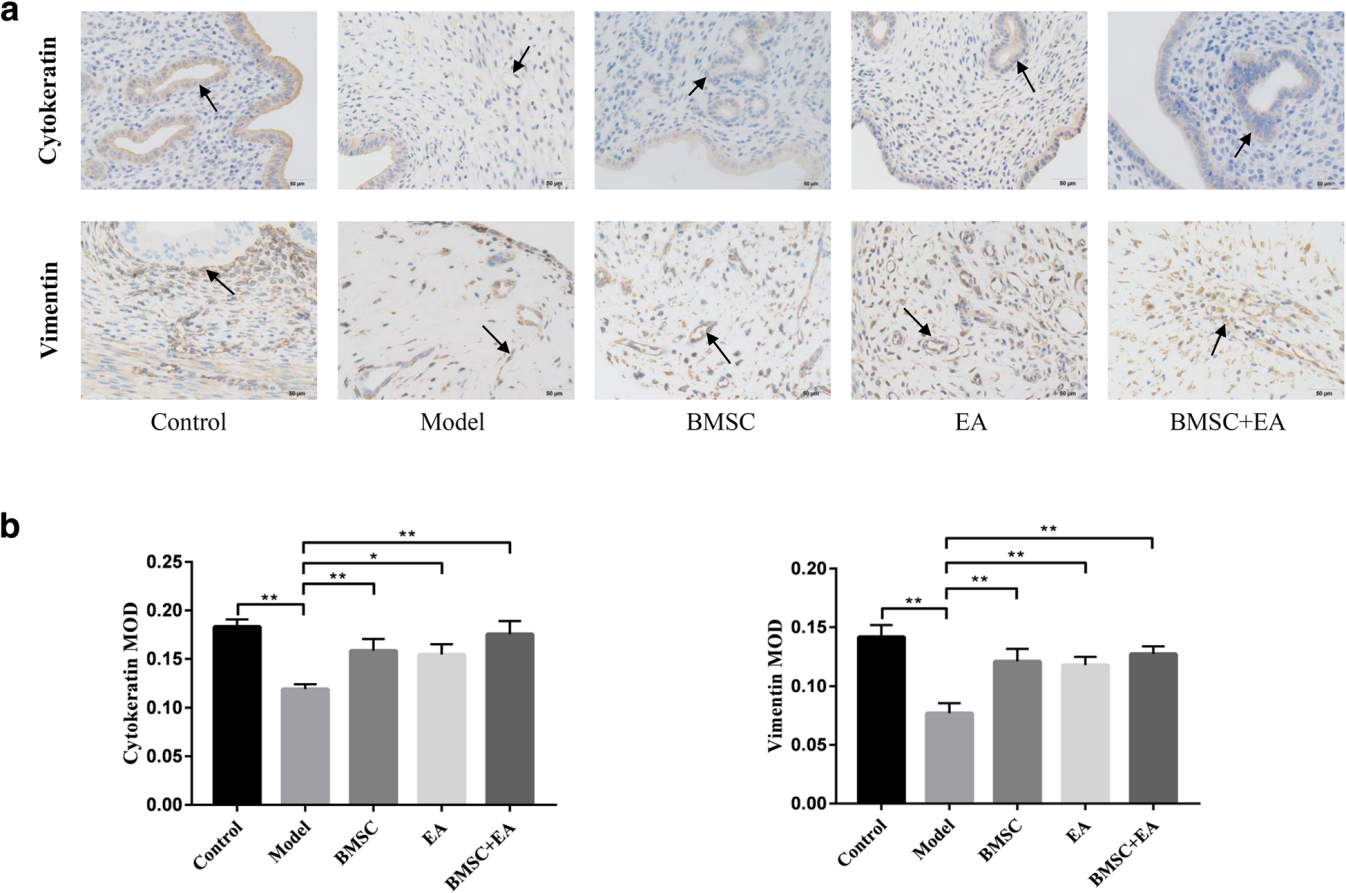
Effects of BMSC transplantation and EA on endometrial epithelial and interstitial cell proliferation. **a** Cytokeratin and vimentin protein expression in each group were detected by immunohistochemistry (× 400), with the arrow indicating the site where brown-yellow particles were deposited. **b** The histogram shows the endometrial cytokeratin and vimentin MOD values of the rats in each group. Bars represent the mean ± standard error (SEM); *n* = 10 per group. *P < 0.05, **P < 0.01

Cytokeratin and vimentin were expressed in the cytoplasm of endometrial epithelial cells and interstitial cells, respectively. They are involved in cell mitosis and cell differentiation, contributing to the integrity and continuity of organelles [31]. To observe the regeneration and distribution of epithelial cells and interstitial cells on endometrial surfaces, the localization of cytokeratin and vimentin was observed in an IHC analysis. The results showed that the MOD values of cytokeratin and vimentin in the model group were much lower than those in the control group (*P* < 0.01). After BMSC transplantation, EA, or the combination of both treatments, the brown-yellow particle deposition with positive staining was observed in endometrial tissue. The MOD values of each intervention group were higher than those in the model group. However, there was no significant difference between the values of the intervention groups (Fig. 4a, b).

**Fig. 5:**
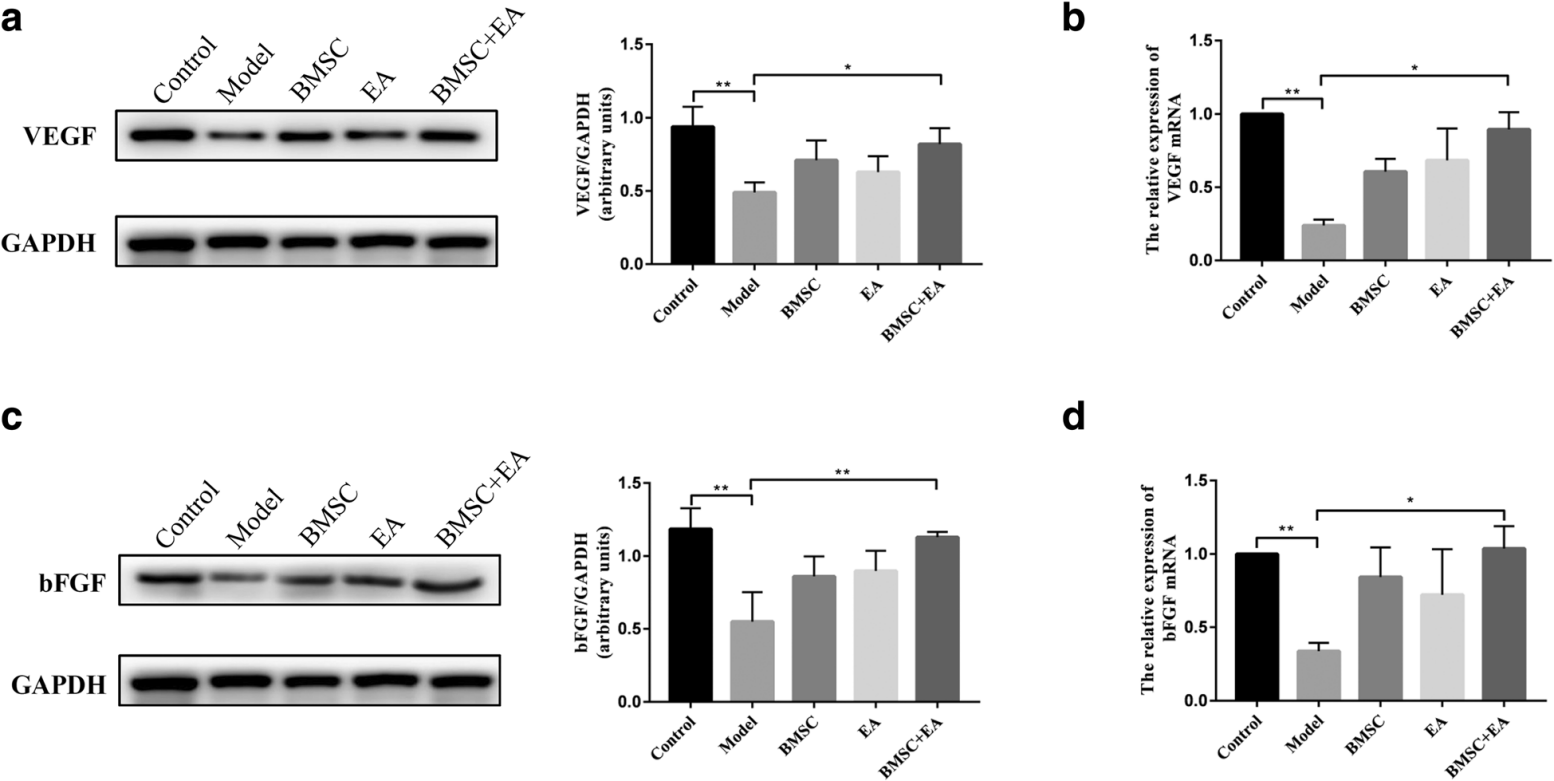
Influence of BMSC transplantation and EA on the release of endometrial surface growth factors. **a** The expression of the vascular endothelial growth factor (VEGF) protein in each group was detected by western blotting. The histogram shows the relative ratio of the expression level of the target protein to that of an internal reference. **b** VEGF mRNA expression in each group was detected by qRT-PCR. R-actin served as an internal reference for the qRT-PCR. **c** The expression of the basic fibroblast growth factor (bFGF) protein in each group was detected by western blotting. The histogram shows the relative ratio of the expression level of the target protein to that of an internal reference. **d** bFGF mRNA expression in each group was detected by qRT-PCR. R-actin served as an internal reference for the qRT-PCR. Bars represent the mean ± standard error (SEM); *n* = 5 per group. *P < 0.05, **P < 0.01

Previous studies have confirmed that BMSCs promoted angiogenesis, repaired tissue damage, and promoted functional remodeling by secreting cytokines, such as VEGF, bFGF, and insulin-like growth factors [32, 33]. As shown by the western blotting and qRT-PCR assay of VEGF and bFGF endometrial surface expression, the expression of VEGF and bFGF on the intimal surface of the injured endometrium was upregulated in both the BMSC-only and EA-only groups, which did not show a significant difference compared with the model group. Intriguingly, VEGF and bFGF expression was significantly upregulated in the BMSC + EA group (*P* < 0.01 and *P* < 0.05, respectively; Fig. 5). These results suggest that EA can enhance the paracrine effect of BMSCs in the injured uterus and that a combination of BMSCs and EA can effectively promote the regeneration of endometrial cells.

### BMSCs combined with EA improves endometrial receptivity and increases embryo implantation rate

**Fig. 6:**
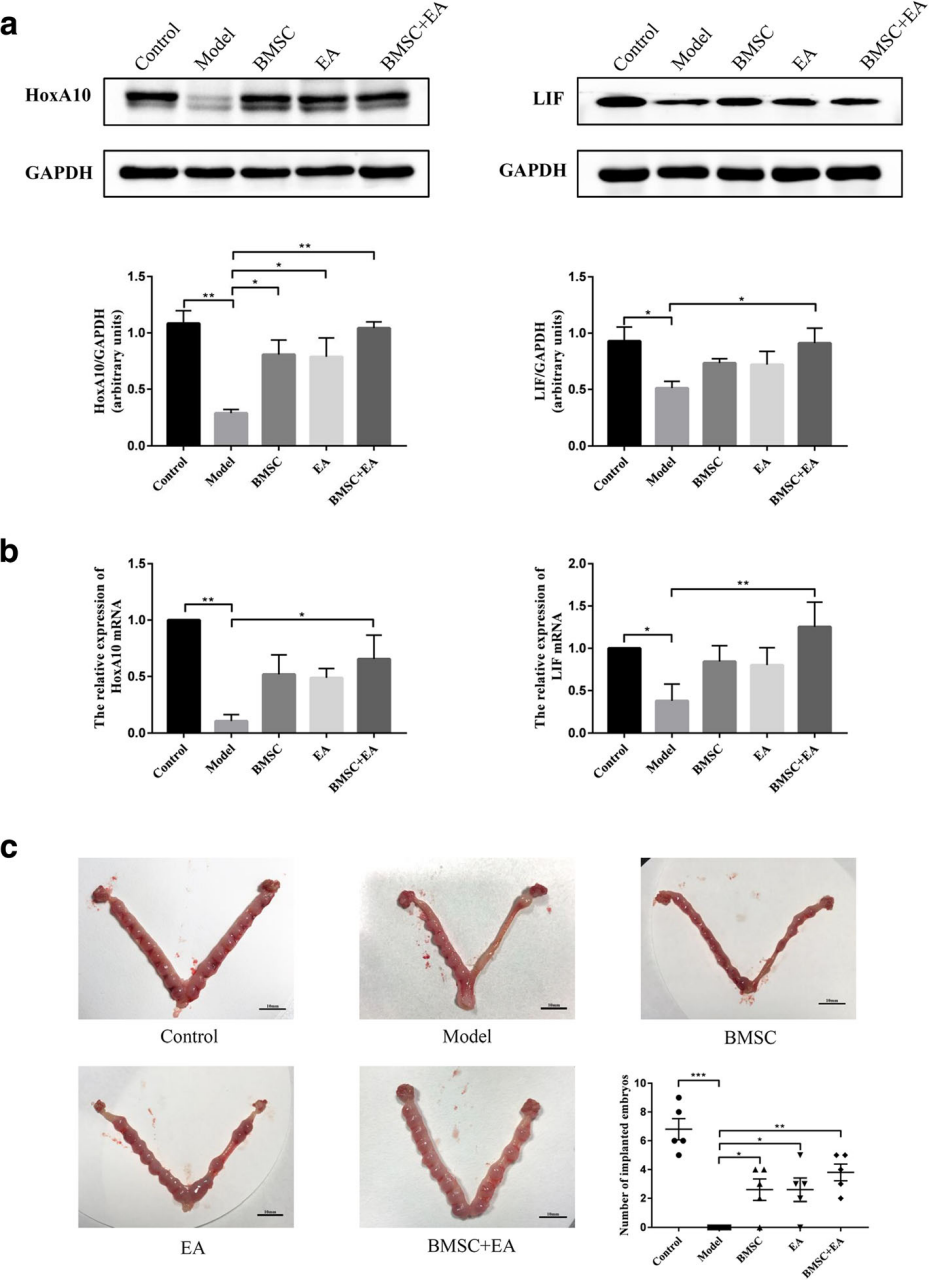
Impact of BMSC transplantation and EA on endometrial receptivity. **a** The expression of Homeobox A10 (HoxA10) and leukemia inhibitory factor (LIF) protein in each group were detected by western blotting. The histogram shows the relative ratio of the expression level of the target protein to that of the internal reference. Bars represent the mean ± SEM; *n* = 5 per group. **b** HoxA10 and LIF mRNA expression in each group were detected by qRT-PCR. R-actin served as an internal reference for the qRT-PCR. Bars represent the mean ± SEM; *n* = 5 per group. **c** Uterine embryo implantation in each group. The scatter plot shows the embryo implantation ratio in the control group, BMSC group, EA group, and BMSC + EA group. Bars represent the mean ± standard error (SEM); *n* = 5 per group. *P < 0.05, **P < 0.01, ***P < 0.001

Endometrial thickness is closely related to the success of embryonic implantation, with a thin endometrium leading to embryo implantation failure or a reduced embryo implantation rate. HoxA10 is a critical regulatory factor in embryo implantation, affecting endometrial decidualization and embryo adhesion by regulating downstream gene expression [34]. LIF is a pleiotropic cytokine secreted by glands that directly target endometrial epithelial cells and affect blastocyst implantation by promoting endometrial receptor expression [35]. To detect the effect of BMSCs and EA on endometrium repair, we examined the expression of marker molecules associated with endometrial receptivity. The western blotting results showed that as compared with the model group, the expression of HoxA10 and LIF were upregulated in all the intervention groups, with a significant increase in the BMSC + EA group. However, there was no statistically significant difference in the HoxA10 and LIF expression among the three intervention groups (Fig. 6a).

As shown by the results of the qRT-PCR of HoxA10 and LIF expression at the mRNA level, HoxA10 and LIF mRNA levels were reduced in the model group as compared with those in the control group. In contrast, significantly upregulated expression of HoxA10 and LIF mRNA was detected in the BMSC + EA group (*P* < 0.05 and *P* < 0.01, respectively). There were no significant differences in HoxA10 and LIF mRNA expression levels between the BMSC-only and EA-only groups (Fig. 6b).

To further investigate the effects of BMSC transplantation and EA on endometrial functional reconstruction, we examined the embryo implantation rate in each group. The number of embryo implantations was highest in the control group (*n* = 5), with 8, 6, 6, 5, and 9 embryos, respectively, with no embryo implantation in the injured uterus of the model group. As compared with the model group, the number of embryo implantations in the BMSC, EA, and BMSC + EA group were increased, which were 0, 4, 3, 4, 2; 0, 5, 2, 3, 3; and 3, 4, 5, 2, 5 (*P* < 0.05, *P* < 0.05, and *P* < 0.01, respectively). The largest numbers of embryos were found in the BMSC + EA group (Fig. 6c).

## Discussion

This study demonstrated that an injection of absolute ethanol in the uterus caused significant injury, with endometrial thinning, decreased numbers of glands and blood vessels, and interstitial edema. Treatment with BMSCs, EA, or a combination of both substantially improved endometrial damage. After the treatments, endometrial interstitial cells, epithelial cells, and vascular density were increased, and cytokine levels were upregulated. These findings demonstrated that CXCR4 expressed by BMSCs specifically bound to SDF-1 released from the damaged endometrium and that BMSCs migrated to the site of damage, further affecting cell regeneration and paracrine function. Moreover, EA effectively mobilized and induced BMSCs to migrate (i.e., homing) to the injured sites in vivo, enhanced the efficacy of stem cell repair damage, improved endometrial receptivity, and increased embryo implantation rates.

Thin or damaged endometrium causes uterine factor-derived infertility, resulting in failed embryonic implantation. Regeneration of the endometrium is a major issue in gynecology and reproductive medicine. To increase the endometrial thickness, various strategies have been adopted, such as systemic or vaginal estrogen [36, 37], low-dose aspirin supplements [38], and intravaginal sildenafil [39], all with limited success. Other methods, such as the use of aromatase inhibitors [40] and delaying the administration of human chorionic gonadotropin [41], have also been suggested. However, these options to improve endometrial thickness are controversial. Cell-based therapies currently using endometrial stem/progenitor cells are expected to be used to restore poor endometrium. However, it is not enough that the damaged endometrium is repaired by endometrial stem/progenitor cell regeneration.

MSCs are a kind of adult stem cells derived from the mesoderm. They are mainly found in connective tissue and organ stroma and are most abundant in the bone marrow [8]. Previous research has showed that transplanted cells can provide morphological and functional benefits through multiple mechanisms, including trophic support, cell replacement, regeneration of endogenous cells, immunosuppression/anti-inflammation, and regulatory interactions with endogenous cells [42]. A clinical investigation has proved that stem cells existing in the endometrium may originate from the bone marrow [43]. A number of experiments have demonstrated that BMSCs appeared to play an important role in endometrial rebuilding [10, 44–49].

However, the effects of MSCs on tissue repair in vivo differ, depending on the affected local microenvironment, especially in the case of endometrial lesions [50]. There are several methods to enhance the effect of MSCs on injury repair: The first method is to combine collagen scaffolds or other biological materials and transplant them directly to the site of injury [51]. The second method is to utilize growth factors, such as heparin-binding epidermal growth factor and VEGF [44, 52]. The third method is to genetically modify MSCs [53]. However, these in vitro modification processes add a number of cumbersome steps, which greatly limit the use of MSCs in clinical practice. Therefore, it is important to find a simple and effective way to enhance the effects of BMSCs.

EA is widely used in traditional Chinese medicine. Due to its few side effects, convenience, and unique impacts on general well-being, acupuncture has gained significant popularity around the world. Acupuncture points are the locations where the pointer can enter some of the deeper tissue components and convey impressions primarily along the meridians. Specific acupuncture points are associated with unique connective tissue locations [23]. In this study, the stimulation points were Sanyinjiao (SP6), Guanyuan (CV4), and Zigong (EX-CA1). According to the principles of traditional Chinese medicine and modern acupoint theory, SP6 is considered a classic acupuncture point for female diseases. It can soften and coordinate liver function and is beneficial to kidney qi. CV4 and EX-CA1 can nourish the uterus and regulate shaft function [54]. Some studies have shown that stimulating these acupuncture points can regulate uterine myoelectric activity and neuroendocrine system and increase endometrial blood flow, thereby improving endometrial receptivity and providing conditions conducive to embryo implantation [17, 55–58]. Based on the favorable regulation of the uterus by EA, we chose SP6, CV4, and EX-CA1 acupoints to explore whether EA was beneficial for stem cells in repairing damage in the uterus. To simulate the clinical treatment of patients with thin endometrium, the EA intervention was administered to rats for three consecutive estrous cycles. The EA type selected was disperse-dense waves, which can promote metabolism and blood circulation, improve tissue nutrition, and eliminate local inflammation and edema [59, 60].

In the present study, we transplanted BMSCs through the tail vein of rats and observed their tissue repair effect on thin endometrium, with or without EA stimulation. Through a histological analysis, we found that both BMSCs alone and EA stimulation alone promoted endometrial thickening. After the treatment, blood vessels and glands regenerated in intimal tissue, and the structure of the uterus was restored to some extent. Further, a combination strategy comprising BMSCs and EA yielded the best results. These findings are consistent with previous research, which found that EA combined with BMSC transplantation promoted spinal cord and intestinal damage repair [61, 62]. We conclude that EA combined with BMSCs appears to be a more effective means of repairing endometrial damage than BMSCs alone. It should be noted that the selected acupuncture points differ, depending on the disease. Besides, the duration of acupuncture and specific mechanisms of action are also different in dealing with different diseases.

In our study, the presence of stem cells labeled with a fluorescent dye was visually detected in both the BMSC and BMSC + EA groups by immunofluorescence. The number of stem cells in the BMSC + EA group was slightly higher than that in the BMSC-only transplantation group. In general, stem cell migration is closely related to the SDF-1/CXCR4 axis [63]. In the present study, western blotting and qRT-PCR also revealed high expression of SDF-1 and CXCR4 after combining BMSCs with EA. These results suggest that EA enhance the chemotaxis of BMSCs by activating the SDF-1/CXCR4 signal axis and promoting the migration of BMSCs to the injured site.

It has been reported that BMSCs played an important role in tissue regeneration through the paracrine production of various growth factors [33]. Hence, we examined the effect of BMSCs and EA on the expression of multiple cytokines in the injured area. Cytokeratin and vimentin maintain organelle integrity and continuity by participating in cell mitosis and cell differentiation [31]. VEGF, an important regulator of vascular morphogenesis, is also involved in endothelial cell proliferation and mobilization, angiogenesis, and remodeling [64]. bFGF is a member of the fibroblast growth factor family that stimulates proliferation and differentiation of endothelial cells, promotes the release of basement membrane-degrading enzymes, and induces the growth of new blood vessels [65]. These cytokines are closely related to tissue regeneration and angiogenesis. In the present study, cytokeratin, vimentin, VEGF, and bFGF expression increased in both the EA and BMSC groups, with a significant increase in the levels in the BMSC + EA group. These findings suggest that EA promotes the paracrine effect of BMSCs at the site of local injury and that it improves the ability of BMSCs to differentiate into cells required for tissue regeneration.

Endometrial receptivity refers to the state in which the endometrium is allowed to locate, adhere, invade, and change the endometrial stroma, which is key to successful embryo implantation. The present study investigated the role of HoxA10 and LIF in endometrial receptivity. HoxA10 is a transcriptional regulatory gene found in Drosophila. It plays an important role in the regulation of embryonic development and cell-directed differentiation and proliferation [66, 67]. LIF is a pleiotropic cytokine secreted by glands that directly targets endometrial epithelial cells. It promotes increased expression of uterine receptor proteins [68] and initiates intimal decidualization by activating signal transducers and activators of transcription 3 [69]. We examined the expression of these two molecules to determine the effects of BMSCs and EA on endometrial receptivity functions. As compared with the control group, all the treatments upregulated endometrial surface expression of HoxA10 and LIF, with the most significant increase observed in the BMSC + EA group. These findings may be explained by beneficial effects of BMSCs and EA on glandular tissue regeneration and restoration of the normal glandular secretion function of the endometrium.

To further investigate the effects of BMSC transplantation and EA on uterine functional reconstruction, we examined the implantation of endometrial embryos in rats. The fertility tests showed that as compared with the model group, the implantation efficiency was enhanced with BMSC transplantation, EA, and a combination of both treatments, with the most effective implantation in the combined treatment group. These results suggest that BMSCs combined with EA improves fertility in rats. Further studies are needed with larger numbers of rats to confirm functional regeneration of the endometrium.

## Conclusion

We conducted a preliminary study about the synergistic effect of BMSCs and EA on endometrial injury. The results suggest that EA enhance the chemotaxis of stem cells, promotes BMSCs transplanted through the tail vein into the uterus, enhances the paracrine effect of stem cells in the damaged uterus, and plays an effective auxiliary role in stem cell repair. This study points to a new method for the clinical application of BMSCs in the treatment of endometrial injury and provides new insights into use of EA combined with BMSCs as a treatment for various types of tissue damage.

## Additional file

Additional file 1:**Table S1.** Endometrial morphology of rats in each group ($\overline{\boldsymbol{x}}\pm \mathbf{s}$). (DOCX 19 kb)

## Data Availability

All data generated and/or analyzed during this study are included in this published article.

## Abbreviations

ART: Assisted reproductive technology
BCA: Bicinchoninic acid
bFGF: Basic fibroblast growth factor
BMSC: Bone marrow mesenchymal stem cell
BSA: Bovine serum albumin
CXCR4: C-X-C chemokine receptor type 4
DAB: Diaminobenzidine
EA: Electro-acupuncture
EDTA: Ethylenediaminetetraacetic acid
ER: Estrogen receptor
FACS: Fluorescence-activated cell sorting
FBS: Fetal bovine serum
GAPDH: Glyceraldehyde 3-phosphate dehydrogenase
H&E: Hematoxylin and eosin
HB-EGF: Heparin-binding epidermal growth factor
hCG: Human chorionic gonadotropin
HoxA10: Homeobox A10
IGF: Insulin-like growth factors
IHC: Immunohistochemistry
IVF-ET: In-vitro fertilization and embryo transfer
L-DMEM: Low glucose Dulbecco’s modified Eagle’s medium
LIF: Leukemia inhibitory factor
MOD: Mean optical density
MSCs: Mesenchymal stem cells
PR: Progesterone receptor
PVDF: Polyvinylidene fluoride
qRT-PCR: Quantitative reverse-transcription polymerase chain reaction
RIPA: Radio-immunoprecipitation assay
SD: Sprague–Dawley
SDF-1: Stromal cell-derived factor-1
SEM: Standard error of mean
SPF: Specific pathogen free
STAT3: Signal transducer and activator of transcription 3
TBST: Tris-buffered saline Tween-20
VEGF: Vascular endothelial growth factor

## References

[1] Shufaro Y, Simon A, Laufer N, Fatum M (2008). Thin unresponsive endometrium--a possible complication of surgical curettage compromising ART outcome. J Assist Reprod Gen..

[2] Bobdiwala S, Farren J, Mitchell-Jones N, Ayim F, Bourne T (2017). OP11.02: Endometrial thickness and its value in triaging women with a pregnancy of unknown location. Ultrasound Obst Gyn.

[3] Fang R, Cai L, Xiong F, Chen J, Yang W, Zhao X (2016). The effect of endometrial thickness on the day of hCG administration on pregnancy outcome in the first fresh IVF/ICSI cycle. Gynecol Endocrinol.

[4] Torry DS, Torry RJ (1997). Angiogenesis and the expression of vascular endothelial growth factor in endometrium and placenta. Am J Reprod Immunol.

[5] Casper RF (2011). It’s time to pay attention to the endometrium. Fertil Steril.

[6] Mahajan N, Sharma S (2016). The endometrium in assisted reproductive technology: how thin is thin?. J Hum Reprod Sci.

[7] Dhinsa BS, Adesida AB (2012). Current clinical therapies for cartilage repair, their limitation and the role of stem cells. Curr Stem Cell Res T.

[8] Marofi F, Vahedi G, Biglari A, Esmaeilzadeh A, Athari SS (2017). Mesenchymal stromal/stem cells: a new era in the cell-based targeted gene therapy of cancer. Front Immunol.

[9] Abumaree MH, Al Jumah MA, Kalionis B, Jawdat D, Al Khaldi A, AlTalabani AA (2013). Phenotypic and functional characterization of mesenchymal stem cells from chorionic villi of human term placenta. Stem Cell Rev.

[10] Gao L, Huang Z, Lin H, Tian Y, Li P, Lin S (2019). Bone marrow mesenchymal stem cells (BMSCs) restore functional endometrium in the rat model for severe Asherman syndrome. Reprod Sci.

[11] Yi KW, Mamillapalli R, Sahin C, Song J, Tal R, Taylor HS (2019). Bone marrow-derived cells or C-X-C motif chemokine 12 (CXCL12) treatment improve thin endometrium in a mouse model. Biol Reprod.

[12] Singh N, Mohanty S, Seth T, Shankar M, Bhaskaran S, Dharmendra S (2014). Autologous stem cell transplantation in refractory Asherman’s syndrome: a novel cell based therapy. J Hum Reprod Sci..

[13] Jo J, Lee YJ (2017). Effectiveness of acupuncture in women with polycystic ovarian syndrome undergoing in vitro fertilisation or intracytoplasmic sperm injection: a systematic review and meta-analysis. Acupunct Med.

[14] Manheimer E, van der Windt D, Cheng K, Stafford K, Liu J, Tierney J (2013). The effects of acupuncture on rates of clinical pregnancy among women undergoing in vitro fertilization: a systematic review and meta-analysis. Hum Reprod Update.

[15] Huang X, Chen L, Xia YB, Xie M, Sun Q, Yao B (2018). Effects of electroacupuncture on luteal regression and steroidogenesis in ovarian hyperstimulation syndrome model rat. Life Sci.

[16] Li CH, Zhao YF, Ji B, Ren XX, Guo MW, Ding XY (2011). Effect of electroacupuncture on the uterine microcirculation in dysmenorrhea rats. Zhen Ci Yan Jiu.

[17] Ma S, Li D, Feng Y, Jiang J, Shen B (2017). Effects of electroacupuncture on uterine morphology and expression of oestrogen receptors in ovariectomised rats. Acupunct Med.

[18] So EW, Ng EH (2010). Acupuncture in reproductive medicine. Womens health.

[19] Zheng CH, Huang GY, Zhang MM, Wang W (2012). Effects of acupuncture on pregnancy rates in women undergoing in vitro fertilization: a systematic review and meta-analysis. Fertil Steril.

[20] Emelyanov AN, Borisova MV, Kiryanova VV (2016). Model acupuncture point: bone marrow-derived stromal stem cells are moved by a weak electromagnetic field. World J Stem Cells.

[21] Salazar TE, Richardson MR, Beli E, Ripsch MS, George J, Kim Y (2017). Electroacupuncture promotes central nervous system-dependent release of mesenchymal stem cells. Stem Cells.

[22] Gao H, Zhao J, Yan-Ping LI (2011). Establishment and identification of rat thin endometrium model. Life Sci Res.

[23] Langevin HM, Yandow JA (2002). Relationship of acupuncture points and meridians to connective tissue planes. Anat Rec.

[24] Guo W, Wang H, Zou S, Gu M, Watanabe M, Wei F (2011). Bone marrow stromal cells produce long-term pain relief in rat models of persistent pain. Stem Cells.

[25] Friedenstein AJ, Chailakhyan RK, Gerasimov UV (1987). Bone marrow osteogenic stem cells: in vitro cultivation and transplantation in diffusion chambers. Cell Tissue Kinet.

[26] Lapidot T, Petit I (2002). Current understanding of stem cell mobilization: the roles of chemokines, proteolytic enzymes, adhesion molecules, cytokines, and stromal cells. Exp Hematol.

[27] Croitoru-Lamoury J, Lamoury FM, Zaunders JJ, Veas LA, Brew BJ (2007). Human mesenchymal stem cells constitutively express chemokines and chemokine receptors that can be upregulated by cytokines, IFN-beta, and Copaxone. J Interf Cytok Res.

[28] Askari AT, Unzek S, Popovic ZB, Goldman CK, Forudi F, Kiedrowski M (2003). Effect of stromal-cell-derived factor 1 on stem-cell homing and tissue regeneration in ischaemic cardiomyopathy. Lancet..

[29] Liu N, Tian J, Cheng J, Zhang J (2013). Migration of CXCR4 gene-modified bone marrow-derived mesenchymal stem cells to the acute injured kidney. J Cell Biochem.

[30] Xu X, Zhu F, Zhang M, Zeng D, Luo D, Liu G, Cui W, Wang S (2013). Stromal cell-derived factor-1 enhances wound healing through recruiting bone marrow-derived mesenchymal stem cells to the wound area and promoting neovascularization. Cells Tissues Organs.

[31] Casado-Vela J, Rodriguez-Suarez E, Iloro I, Ametzazurra A, Alkorta N, Garcia-Velasco JA (2009). Comprehensive proteomic analysis of human endometrial fluid aspirate. J Proteome Res.

[32] Lai RC, Arslan F, Lee MM, Sze NS, Choo A, Chen TS (2010). Exosome secreted by MSC reduces myocardial ischemia/reperfusion injury. Stem Cell Res.

[33] van Poll D, Parekkadan B, Cho CH, Berthiaume F, Nahmias Y, Tilles AW (2008). Mesenchymal stem cell-derived molecules directly modulate hepatocellular death and regeneration in vitro and in vivo. Hepatology..

[34] Zanatta A, Rocha AM, Carvalho FM, Pereira RM, Taylor HS, Motta EL (2010). The role of the Hoxa10/HOXA10 gene in the etiology of endometriosis and its related infertility: a review. J Assist Reprod Gen..

[35] Stewart CL, Kaspar P, Brunet LJ, Bhatt H, Gadi I, Kontgen F (1992). Blastocyst implantation depends on maternal expression of leukaemia inhibitory factor. Nature..

[36] Gerli S, Gholami H, Manna C, Di Frega AS, Vitiello C, Unfer V (2000). Use of ethinyl estradiol to reverse the antiestrogenic effects of clomiphene citrate in patients undergoing intrauterine insemination: a comparative, randomized study. Fertil Steril.

[37] Cetinkaya K, Kadanali S (2012). The effect of administering vaginal estrogen to clomiphene citrate stimulated cycles on endometrial thickness and pregnancy rates in unexplained infertility. J Turk Ger Gynecol Assoc.

[38] Check JH, Dietterich C, Lurie D, Nazari A, Chuong J (1998). A matched study to determine whether low-dose aspirin without heparin improves pregnancy rates following frozen embryo transfer and/or affects endometrial sonographic parameters. J Assist Reprod Gen.

[39] Sher G, Fisch JD (2000). Vaginal sildenafil (Viagra): a preliminary report of a novel method to improve uterine artery blood flow and endometrial development in patients undergoing IVF. Hum Reprod.

[40] Badawy A, Mosbah A, Shady M (2008). Anastrozole or letrozole for ovulation induction in clomiphene-resistant women with polycystic ovarian syndrome: a prospective randomized trial. Fertil Steril.

[41] Dickey RP, Olar TT, Taylor SN, Curole DN, Matulich EM (1993). Relationship of endometrial thickness and pattern to fecundity in ovulation induction cycles: effect of clomiphene citrate alone and with human menopausal gonadotropin. Fertil Steril.

[42] Cervello I, Gil-Sanchis C, Santamaria X, Cabanillas S, Diaz A, Faus A (2015). Human CD133(+) bone marrow-derived stem cells promote endometrial proliferation in a murine model of Asherman syndrome. Fertil Steril.

[43] Taylor HS (2004). Endometrial cells derived from donor stem cells in bone marrow transplant recipients. Jama..

[44] Jing Z, Yi Y, Xi H, Sun LQ, Yanping L (2018). Therapeutic effects of VEGF gene-transfected BMSCs transplantation on thin endometrium in the rat model. Stem Cells Int.

[45] Yang H, Wu S, Feng R, Huang J, Liu L, Liu F (2017). Vitamin C plus hydrogel facilitates bone marrow stromal cell-mediated endometrium regeneration in rats. Stem Cell Res Ther.

[46] Wang J, Ju B, Pan C, Gu Y, Zhang Y, Sun L (2016). Application of bone marrow-derived mesenchymal stem cells in the treatment of intrauterine adhesions in rats. Cell Physiol Biochem.

[47] Cong Q, Li B, Wang Y, Zhang W, Cheng M, Wu Z (2014). In vitro differentiation of bone marrow mesenchymal stem cells into endometrial epithelial cells in mouse: a proteomic analysis. Int J Clin Exp Patho.

[48] Zhao Jing, Zhang Qiong, Wang Yonggang, Li Yanping (2014). Uterine Infusion With Bone Marrow Mesenchymal Stem Cells Improves Endometrium Thickness in a Rat Model of Thin Endometrium. Reproductive Sciences.

[49] Zhang WB, Cheng MJ, Huang YT, Jiang W, Cong Q, Zheng YF (2012). A study in vitro on differentiation of bone marrow mesenchymal stem cells into endometrial epithelial cells in mice. Eur J Obstet Gyn R B.

[50] Ding L, Li X, Sun H, Su J, Lin N, Peault B (2014). Transplantation of bone marrow mesenchymal stem cells on collagen scaffolds for the functional regeneration of injured rat uterus. Biomaterials..

[51] Cao Y, Sun H, Zhu H, Zhu X, Tang X, Yan G (2018). Allogeneic cell therapy using umbilical cord MSCs on collagen scaffolds for patients with recurrent uterine adhesion: a phase I clinical trial. Stem Cell Res Ther.

[52] Watkins DJ, Yang J, Matthews MA, Besner GE (2013). Synergistic effects of HB-EGF and mesenchymal stem cells in a murine model of intestinal ischemia/reperfusion injury. J Pediatr Surg.

[53] Liu X, Zuo D, Fan H, Tang Q, Shou Z, Cao D (2014). Over-expression of CXCR4 on mesenchymal stem cells protect against experimental colitis via immunomodulatory functions in impaired tissue. J Mol Histol.

[54] Xia L, Xia Y (2018). Clinical research and the effect mechanism on premature ovarian failure treated with acupuncture in recent 20 years. Zhongguo Zhen Jiu.

[55] Chen L, Sun HX, Xia YB, Sui LC, Zhou J, Huang X (2016). Electroacupuncture decreases the progression of ovarian hyperstimulation syndrome in a rat model. Reprod BioMed Online.

[56] Manneras L, Jonsdottir IH, Holmang A, Lonn M, Stener-Victorin E (2008). Low-frequency electro-acupuncture and physical exercise improve metabolic disturbances and modulate gene expression in adipose tissue in rats with dihydrotestosterone-induced polycystic ovary syndrome. Endocrinology..

[57] Sun J, Zhao JM, Ji R, Liu HR, Shi Y, Jin CL (2013). Effects of electroacupuncture of “Guanyuan” (CV 4)-“Zhongji” (CV 3) on ovarian P450 arom and P450c 17alpha expression and relevant sex hormone levels in rats with polycystic ovary syndrome. Zhen Ci Yan Jiu.

[58] Stener-Victorin E, Jedel E, Janson PO, Sverrisdottir YB (2009). Low-frequency electroacupuncture and physical exercise decrease high muscle sympathetic nerve activity in polycystic ovary syndrome. Am J Physiol Regul Integr Comp Physiol.

[59] Sun J, Liang Y, Wang C, Shao X, Fang J (2017). Clinical experience of acupuncture and moxibustion in the diagnosis and treatment of persistent somatoform pain disorder. Zhongguo Zhen Jiu.

[60] Wang WB, Yang LF, He QS, Li T, Ma YY, Zhang P (2016). Mechanisms of electroacupuncture effects on acute cerebral ischemia/reperfusion injury: possible association with upregulation of transforming growth factor beta 1. Neural Regen Res.

[61] Liu Z, Ding Y, Zeng YS (2011). A new combined therapeutic strategy of governor vessel electro-acupuncture and adult stem cell transplantation promotes the recovery of injured spinal cord. Curr Med Chem.

[62] Geng Y, Chen D, Zhou J, Lu J, Chen M, Zhang H (2016). Synergistic effects of electroacupuncture and mesenchymal stem cells on intestinal ischemia/reperfusion injury in rats. Inflammation..

[63] Jin W, Liang X, Brooks A, Futrega K, Liu X, Doran MR (2018). Modelling of the SDF-1/CXCR4 regulated in vivo homing of therapeutic mesenchymal stem/stromal cells in mice. Peer J.

[64] Sharkey AM, Day K, McPherson A, Malik S, Licence D, Smith SK (2000). Vascular endothelial growth factor expression in human endometrium is regulated by hypoxia. J Clin Endocr Metab.

[65] Achache H, Revel A (2006). Endometrial receptivity markers, the journey to successful embryo implantation. Hum Reprod Update.

[66] McGinnis W, Garber RL, Wirz J, Kuroiwa A, Gehring WJ (1984). A homologous protein-coding sequence in Drosophila homeotic genes and its conservation in other metazoans. Cell..

[67] Scott MP, Weiner AJ (1984). Structural relationships among genes that control development: sequence homology between the Antennapedia, Ultrabithorax, and fushi tarazu loci of Drosophila. P Natl Acad Sci USA.

[68] Aghajanova L (2010). Update on the role of leukemia inhibitory factor in assisted reproduction. Curr Opin Obstet Gyn.

[69] Shuya LL, Menkhorst EM, Yap J, Li P, Lane N, Dimitriadis E (2011). Leukemia inhibitory factor enhances endometrial stromal cell decidualization in humans and mice. PLoS One.

