# Correction: The synergistic effect of electroacupuncture and bone mesenchymal stem cell transplantation on repairing thin endometrial injury in rats

**DOI:** 10.1186/s13287-023-03421-5

**Published:** 2023-08-08

**Authors:** Liangjun Xia, Qingyu Meng, Jin Xi, Qin Han, Jie Cheng, Jie Shen, Youbing Xia, Liyun Shi

**Affiliations:** 1 School of Medicine and Life Science, Nanjing University of Chinese Medicine, Nanjing, 210046 China; 2 Xuzhou Medical University, Xuzhou, 221004 China; 3 The Second Clinical College, Nanjing University of Chinese Medicine, Nanjing, 210046 China

**Correction: Stem Cell Research & Therapy (2019) 10:244** 10.1186/s13287-019-1326-6

In the original article, the authors found an error in Fig. 5c. Specifically, the image for GAPDH blot in Fig. 5a was repeatedly inserted in Fig. 5c during the multi-panel figure assembly. The correct image of GAPDH in Fig. 5c has now been provided and the authors apologize for the mistake made. This correction does not change the results or conclusion of the original study.

**Fig. 5:**
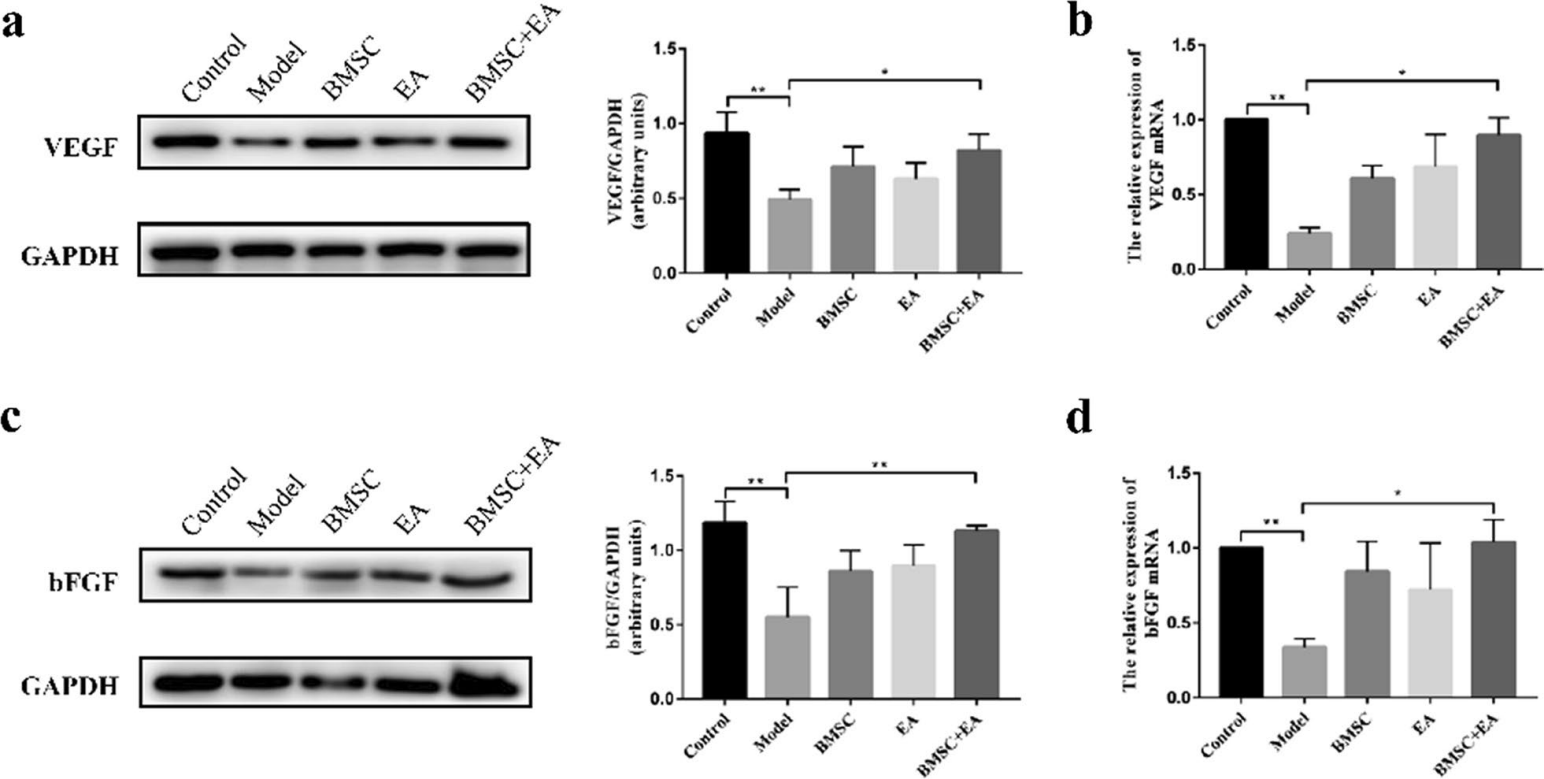
Influence of BMSC transplantation and EA on the release of endometrial surface growth factors. **a** The expression of the vascular endothelial growth factor (VEGF) protein in each group was detected by western blotting. The histogram shows the relative ratio of the expression level of the target protein to that of an internal reference. **b** VEGF mRNA expression in each group was detected by qRT-PCR. R-actin served as an internal reference for the qRT-PCR. **c** The expression of the basic fibroblast growth factor (bFGF) protein in each group was detected by western blotting. The histogram shows the relative ratio of the expression level of the target protein to that of an internal reference. **d** bFGF mRNA expression in each group was detected by qRT-PCR. R-actin served as an internal reference for the qRT-PCR. Bars represent the mean ± standard error (SEM); *n* = 5 per group. *P < 0.05; **P < 0.01
