# Supplementary material for: The synergistic effect of electroacupuncture and bone mesenchymal stem cell transplantation on repairing thin endometrial injury in rats
Source: Stem Cell Res Ther. 2019 Aug 7;10:244. doi: 10.1186/s13287-019-1326-6 (PMC6686409; doi:10.1186/s13287-019-1326-6)
Supplement: Supplementary file 1 — Table S1. Endometrial morphology of rats in each group ($\overline{\boldsymbol{x}}\pm \mathbf{s}$x¯±s). (DOCX 19 kb) [file 13287_2019_1326_MOESM1_ESM.docx]

**Table S1** Endometrial morphology of rats in each group（**$\overline{x}$±s**）

| Groups | Endometrium thickness (μm) | Number of glands | Number of blood vessels |
| --- | --- | --- | --- |
| Control | 407.30±64.33** | 2.53±1.02** | 4.05±1.52** |
| Model | 285.23±59.24 | 0.95±0.62 | 1.73±1.00 |
| BMSCs | 371.35±97.47* | 1.73±0.91 | 3.68±0.87* |
| EA | 358.60±64.51* | 1.68±0.91 | 3.70±1.69** |
| Combined | 384.59±86.07** | 1.85±0.88** | 3.85±1.60** |

*n* = 10 per group. **P* <0.05, ***P* <0.01.
